# Adjusting Hospital Reimbursement to Account for Social Influences on Health

**DOI:** 10.1001/jamanetworkopen.2019.13630

**Published:** 2019-10-02

**Authors:** Matthew M. Davis, Kristin Kan

**Affiliations:** Division of Academic General Pediatrics and Primary Care, Department of Pediatrics, Northwestern University Feinberg School of Medicine, Chicago, Illinois; Departments of Medicine, Medical Social Sciences, and Preventive Medicine, Northwestern University Feinberg School of Medicine, Chicago, Illinois; Mary Ann and J. Milburn Smith Child Health Research, Outreach and Advocacy Center, Stanley Manne Children’s Research Institute, Ann and Robert H. Lurie Children’s Hospital of Chicago, Chicago, Illinois; Division of Academic General Pediatrics and Primary Care, Department of Pediatrics, Northwestern University Feinberg School of Medicine, Chicago, Illinois; Mary Ann and J. Milburn Smith Child Health Research, Outreach and Advocacy Center, Stanley Manne Children’s Research Institute, Ann and Robert H. Lurie Children’s Hospital of Chicago, Chicago, Illinois

The strength of the social safety net matters most when the risk to individuals is highest. Acute illnesses and injuries requiring hospital-level care are some of the greatest health risks that children and adults face. In the United States, 2 of the oldest social safety net programs are Medicare and Medicaid, which together now constitute 37% of national health expenditures compared with 34% for private health insurance.^1^ Medicare and Medicaid have endured for more than 50 years, despite political efforts to constrain growth in government spending, in large part because they cover the costs of hospital care that can be economically catastrophic for individuals and families.

Medicare and Medicaid also function as a safety net for hospitals by reimbursing institutions for the expenses of hospitalizations not paid by patients themselves. In the case of children’s hospitals that provide care to large numbers of patients with Medicaid coverage, such reimbursement often falls short of covering the hospitals’ care-delivery costs.^2^ This shortfall is the rationale for the Medicaid Disproportionate Share Hospital (DSH) payment policy, which enables states to work with the federal government to provide payments to hospitals to offset the costs of uncompensated and undercompensated inpatient care. Even accounting for DSH payments, the costs of providing inpatient care for children on Medicaid often exceed reimbursement, creating a chronic strain for hospitals with a mission to care for the underserved.^3^

Moreover, hospitals are increasingly aware that children who rely on Medicaid also often experience a myriad of socioeconomic challenges that compound their health care needs. Youth with unstable housing, food insecurity, and/or poor access to timely primary care may present with more severe acute illnesses than patients with more resources and better access to care. Inpatients whose home circumstances are fragile may also require more time and support for their care teams to make postdischarge arrangements, such as home nurse visits, to assure a smooth transition to home.

Differences in socioenvironmental circumstances and their effects on inpatient care are the focus of the study by Michel et al.^4^ The investigators analyzed administrative data from hospitals across France for children aged 28 days to 17 years during a 3-year period (2012–2014).^4^ They characterized each hospitalized child according to the social disadvantage they experienced in their neighborhoods, using an indicator based on the 4 following factors: median household income, unemployment rate, proportion of blue-collar workers in the employed population, and proportion of high school graduates 15 years or older in the population.^5^

Among the more than 4.1 million hospitalizations in the study, children who lived in areas with the greatest socioeconomic disadvantage were significantly more likely to have conditions characterized as intermediate or severe, and their lengths of stay were significantly longer.^4^ Controlling for patient factors, illness characteristics (including severity), and hospital characteristics, overall length of stay was 3% longer for children from the most disadvantaged quintile.^4^ Michel et al^4^ also found that hospitals that had a patient mix with 20% to 60% of patients from the 2 most disadvantaged quintiles were more likely to have a negative financial balance compared with hospitals that served smaller proportions of children from the most disadvantaged quintiles.

These findings, and similar results in analyses of hospitalizations for disadvantaged children receiving Medicaid in the United States,^2^ raise fundamental questions about how better to address the broader social needs of children who require hospitalization. Literature is rapidly emerging about how to address social determinants of health for children through a wide variety of clinical interventions.^6^ We suggest a set of policy alternatives for addressing the challenges of hospital reimbursement for socioeconomically disadvantaged youth.

The current DSH program in the United States is a helpful but ultimately blunt policy approach that provides hospitals with lump-sum payments that are frequently disconnected from the volumes of patients served or the specific costs of care for individual children. It would be more efficient to implement policies that follow the person.

The first option would be to increase Medicaid reimbursement for hospitalizations. Given that Medicaid eligibility for children is predominantly driven by household income, this would serve as a direct adjustment of the level of reimbursement for the numbers of disadvantaged children that a hospital serves. As straightforward as this option would be, it may also be the least politically feasible because of the broad increase in program expenditures from the federal and state governments that would be anticipated with higher reimbursement rates for inpatient stays.

A second option would be to apply a per-patient payment adjustment for geographically defined social risk that is similar to the use of an ecological index of disadvantage in the study by Michel et al.^4^ In this approach, risk adjustment would account for the constraints hospitals located in underresourced areas face when addressing patients’ social needs. For example, Blustein et al^7^ found that hospitals located in counties with chronic poverty or counties that have been designated as having shortages of health care professionals had significantly worse performance on measures included in Medicare’s Value-Based Purchasing program than hospitals with fewer local socioeconomic challenges. Therefore, an enhanced distribution of reimbursements might more equitably support hospitals that disproportionately serve pediatric patients in disadvantaged geographic areas. However, skeptics might be concerned that such a policy would reimburse hospitals without requiring that they attend to hospital-to-hospital differences in quality and outcomes that might also be associated with social determinants of health.

Therefore, a third potential policy remedy would be a per-patient payment adjustment for patients’ social risks, deliberately connected to the quality of patient outcomes. This approach, recommended by a National Academy of Medicine Committee for Medicare, would require separate reporting of quality measures for hospitals in different categories related to distinct levels of social risk in the populations they serve and would include an additional financial incentive for quality improvement.^8^ Under this policy, hospitals could be rewarded for incremental improvements in quality measures against their own historical benchmarks. The payment adjustment could also promote closing gaps in the quality of care that may be worse among institutions serving disadvantaged populations.^9^

A fourth policy option to remedy the underreimbursement of hospitals for the care of children with social disadvantage would encourage hospitals to connect strongly to their roles as anchor institutions in the disadvantaged communities they serve. Anchor institutions are organizations—in health care and other sectors—that commit themselves to hiring, procuring, and investing in disadvantaged communities.^10^ Under this policy alternative, hospitals that achieve specific levels of activities in communities regarding these key domains would earn enriched reimbursement for the children they serve from those same or similarly disadvantaged communities. This particular policy option is appealing because it encourages hospitals to commit to their natural roles as major employers and purchasers of goods and services in their communities while also trying to address systemic poverty and improve employment and investment in the neighborhoods in which their patients live.

These types of policy options would address emerging concerns on both sides of the Atlantic related to the persistent financial vulnerability of hospitals that serve large numbers of children with socioeconomic disadvantage. In the United States, by moving beyond DSH to programs that follow the patient or even follow into the community, there are promising opportunities to address persistent shortfalls in hospital reimbursement for the neediest children while also promoting high-quality care, community engagement, and neighborhood reinvestment.

## References

[1] Centers for Medicare & Medicaid Services. NHE fact sheet. https://www.cms.gov/research-statistics-data-and-systems/statistics-trends-and-reports/nationalhealthexpenddata/nhe-fact-sheet.html. Accessed September 16, 2019.

[2] Colvin JD, Hall M, Berry JG, Financial loss for inpatient care of Medicaid-insured children. JAMA Pediatr. 2016;170(11):1055–1062. doi:10.1001/jamapediatrics.2016.1639 27618284

[3] Davis MM, Kan K. Medicaid and children’s hospitals: a vital but strained double helix for children’s health care. JAMA Pediatr. 2016;170(11):1043–1045. doi:10.1001/jamapediatrics.2016.2328 27617592

[4] Michel M, Alberti C, Carel J-C, Chevreul K. Association of pediatric inpatient socioeconomic status with hospital efficiency and financial balance. JAMA Netw Open. 2019;2(10):e1913656. doi:10.1001/jamanetworkopen.2019.13656 31626320 PMC6813670

[5] Rey G, Jougla E, Fouillet A, Hémon D. Ecological association between a deprivation index and mortality in France over the period 1997–2001: variations with spatial scale, degree of urbanicity, age, gender and cause of death. BMC Public Health. 2009;9:33. doi:10.1186/1471-2458-9-33 19161613 PMC2637240

[6] Beck AF, Cohen AJ, Colvin JD, Perspectives from the Society for Pediatric Research: interventions targeting social needs in pediatric clinical care. Pediatr Res. 2018;84(1):10–21. doi:10.1038/s41390-018-0012-1 29795202

[7] Blustein J, Borden WB, Valentine M. Hospital performance, the local economy, and the local workforce: findings from a US National Longitudinal Study. PLoS Med. 2010;7(6):e1000297. doi:10.1371/journal.pmed.1000297 20613863 PMC2893955

[8] National Academies of Sciences, Engineering, and Medicine. Accounting for Social Risk Factors in Medicare Payment: Identifying Social Risk Factors. Washington, DC: National Academies Press; 2016. 26844313

[9] Casalino LP, Elster A, Eisenberg A, Lewis E, Montgomery J, Ramos D. Will pay-for-performance and quality reporting affect health care disparities? Health Aff (Millwood). 2007;26(3):w405–w414. doi:10.1377/hlthaff.26.3.w405 17426053

[10] Norris T, Howard T. Can hospitals heal America’s communities? https://democracycollaborative.org/content/can-hospitals-heal-americas-communities-0. Accessed September 20, 2019.

